# 616. Candidemia in a Dominican tertiary care hospital: Six years of history

**DOI:** 10.1093/ofid/ofad500.682

**Published:** 2023-11-27

**Authors:** Rita A Rojas-Fermin, Anel E Guzman-Marte, Ann Stephany Sanchez-Marmolejos, Maria Fernanda Cedeño-Bruzual, Javier Rojas-Jiménez, Marlon Rojas -Jiménez

**Affiliations:** Hospital General de la Plaza de la Salud, Santo Domingo, Distrito Nacional, Dominican Republic; Hospital General de la Plaza de la Salud, Santo Domingo, Distrito Nacional, Dominican Republic; Hospital General de la Plaza de la Salud, Santo Domingo, Distrito Nacional, Dominican Republic; Hospital General de la Plaza de la Salud, Santo Domingo, Distrito Nacional, Dominican Republic; Universidad Nacional Pedro Henriquez Ureña, Santo Domingo, Distrito Nacional, Dominican Republic; Universidad Nacional Pedro Henriquez Ureña, Santo Domingo, Distrito Nacional, Dominican Republic

## Abstract

**Background:**

Despite the high mortality caused by invasive candidiasis or the fact that, as of 2022, a list of priority fungal agents was created by WHO, including Candida species in the three categories, global information is not robust, so it is difficult to estimate the distribution of these infections, species, antifungal resistance patterns, and clinical characteristics of the different types of patients affected globally.

**Methods:**

We performed a retrospective review of patients with candidemia from 2017 to 2022. Data extracted from e-medical records from a teaching hospital with 289 beds included clinical and demographic information, co-morbidities, risk factors, antifungal resistance patterns, and outcomes.

**Results:**

A positive rate of 0.75 percent was seen in 210 cases in 197 patients; 103 of them (52.28%) were men. With 52 cases each, 2021 and 2022 saw the most cases. In six years, the average age was 53 (IQR 54). The most frequent comorbidities were hypertension (52.79%), chronic pulmonary disease (45.68%), and nephropathy (31.98%). Intensive Care Unit stays (74.11%) and prior antibiotic exposure (92.89%) were the most prevalent risk factors, with statistical significance for length of stay in the ICU, the use of vasopressors, and central catheters (p 0.001). Surviving patients had a median length of stay of 26 days (p 0.001). The mortality rate for all patients was 49.2%. *C. auris* or C. *krusei* species were not isolated during the study period. The most frequently isolated species were *C*. *tropicalis* (40.4%), followed by *C. parapsilopsis* (23.8%) and *C*. *albicans* (20%). Fluconazole resistance was 6% (n=12) across all isolated species, with *C. parapsilopsis* having the highest prevalence 12% (n=6). *C. tropicalis* was the species most associated with death, followed by *C. albicans*.

**Figure:**
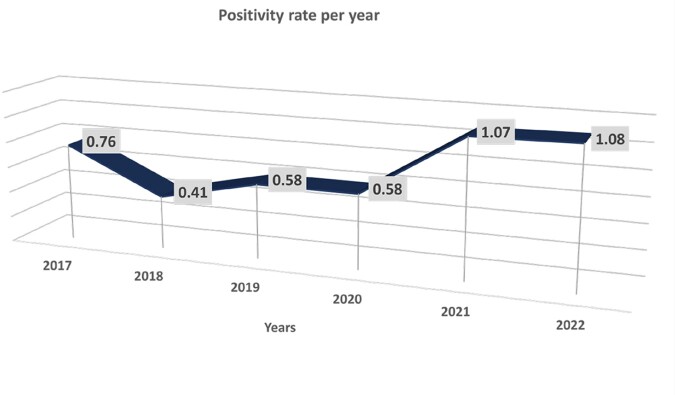

**Figure:**
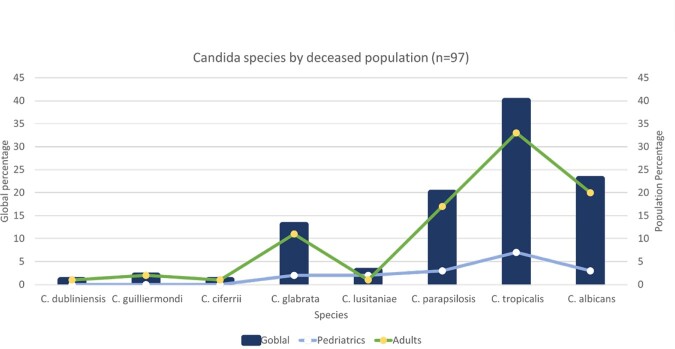

**Figure:**
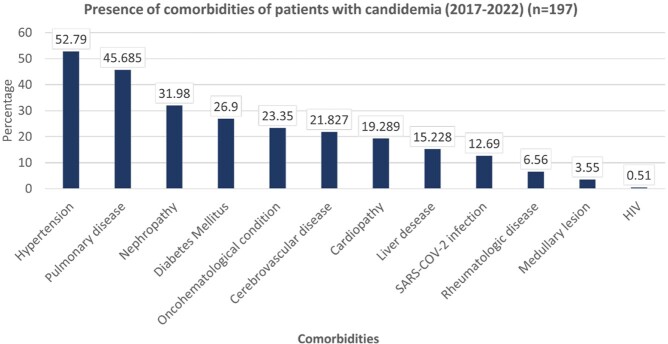

**Conclusion:**

In the post-pandemic period, we observed an increase in *Candida* isolates and noted a steady increase in cases of *C*. *glabrata,* especially in the ICU. Length of ICU stay, vasopressor use, and central catheters were seen as significant risk factors for candidemia, with older patients being the most affected as well as having higher mortality rates. Although *C. auris* hasn´t been detected at our institution, we recommend continued surveillance for our infection prevention program.

**Figure:**
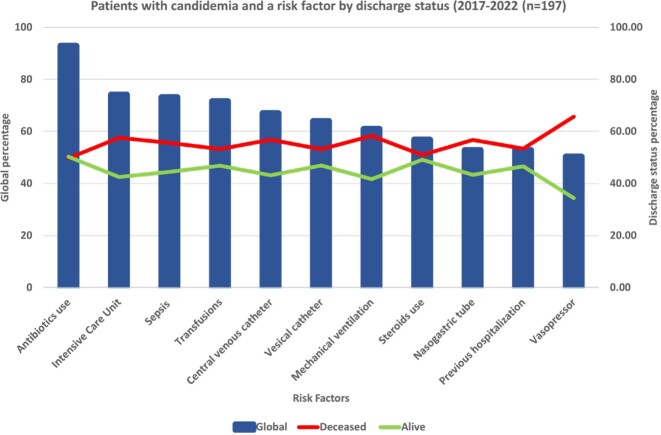

**Figure:**
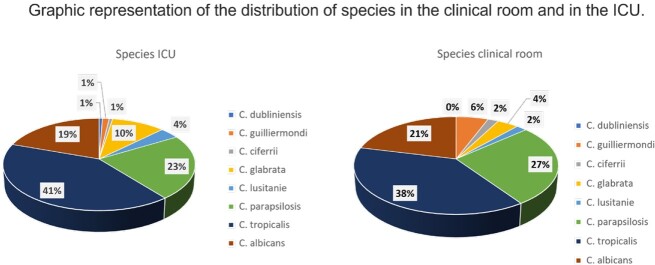

**Disclosures:**

**All Authors**: No reported disclosures

